# Exploring the functional food potential of wild rice (*Zizania palustris*): A comparative analysis of morphological, nutritional, bioactive compounds, and antioxidant activity in whole, milled, and bran fractions

**DOI:** 10.1016/j.fochx.2026.103961

**Published:** 2026-05-08

**Authors:** Xin-Yu Li, Yi-Tong Zhang, Yu-Jie Wang, Sheng-Yi Wang, Yong-Li Wang, Ya-Li Liu, Chu-Yun Wu, Jian-Ya Qian, Chen Zhang

**Affiliations:** School of Food Science and Engineering, Yangzhou University, Huayang Xilu 196, Yangzhou, Jiangsu 225127, People's Republic of China

**Keywords:** Wild rice, Milling fractions, Nutritional composition, Bioactive compounds, Antioxidant activity

## Abstract

This study compared morphological, nutritional, and functional properties of wild rice (*Zizania palustris*, ZP) fractions (whole, milled grain, bran) with white (WR) and brown rice (BR). Morphological properties were investigated by SEM, nutritional composition, amino acid and mineral profiles, antioxidant activities were analyzed using proximate analysis and microplate reader-based colorimetric assays. ZP exhibited elongated kernels with dense endosperm and horseshoe-shaped morphology, with significantly higher protein, dietary fiber, and essential minerals than WR and BR in all fractions, especially bran. ZP bran showed 2–11 fold higher phenolics and flavonoids, and 2–7 fold higher antioxidant activity than rice controls. ZP presented a nutritionally balanced amino acid profile, with higher lysine and essential to total amino acid ratio (∼37%) approaching FAO/WHO recommendations, and unsaturated fatty acids (>87%), including long-chain polyunsaturated fatty acids rarely reported in common cereals. These findings highlight wild rice as a functional food ingredient with enhanced nutritional and antioxidant profiles.

## Highlights

• Wild rice (ZP) has dense endosperm，thick brown bran and a horseshoe-shaped kernel.
• ZP has higher protein, dietary fiber, minerals, and lower starch than white and brown rice.
• ZP has a balanced amino acid profile with 2.3-fold higher lysine than rice controls.
• ZP containes over 86% of unsaturated fatty acids, including rare long-chain PUFAs.
• ZP has 2–11 fold higher phenolics/flavonoids and antioxidant activities, even after milling.

## Graphical abstract

**Figure:**
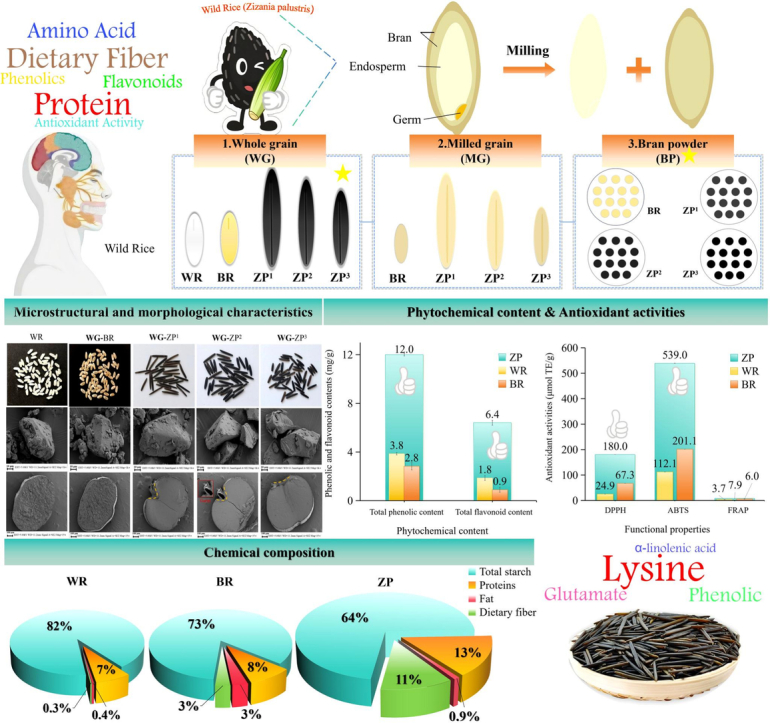

## 1 Introduction

Whole grains are widely recognized as essential sources of various nutrients and phytochemicals, and epidemiological evidence has consistently demonstrated that regular consumption reduces the risk of chronic diseases, such as colorectal cancer, type 2 diabetes mellitus, and cardiovascular disorders (Saleh et al., 2019; Tullio et al., 2021; Wei et al., 2024). Consequently, dietary guidelines worldwide recommend increased whole grain intake. While extensive research has focused on common whole grains (e.g., brown rice or whole wheat), the compositional and functional profiles of many nutritionally promising yet underutilized cereals remain insufficiently characterized, limiting their potential application.

Among these underutilized cereals, wild rice (genus *Zizania*), an aquatic cereal grain primarily cultivated in North America and East Asia, has garnered increasing attention for its unique nutritional properties and health benefits. It is also known as Canadian rice, water oats, or “Jiaobai”, where the culm is consumed as a vegetable following infection by *Ustilago esculenta* in China (Lu et al., 2022). The genus of wild rice includes four species, with *Zizania palustris* and *Zizania aquatica* being the primary species cultivated for human consumption. Although its dark brown bran layer superficially resembles pigmented *Oryza* rice (e.g., black or brown rice), wild rice belongs to a different genus and possesses distinct genetic, morphological, and compositional profiles.

Recognized as a whole grain by the U.S. Food and Drug Administration (FDA) since 2006, wild rice has a nutritional profile distinct from conventional cereals. For example, its protein content is approximately twice that of white rice (Yang et al., 2025) and brown rice (Surendiran et al., 2014; Summpunn et al., 2023), while dietary fiber content is 3–8 times higher (Massaretto et al., 2023; Peres et al., 2023), contributing to a slower carbohydrate digestion rate and beneficial for blood glucose management (Han et al., 2013; Kumar & Prabhasankar, 2014). Furthermore, numerous studies have demonstrated that wild rice possesses significant health benefits, including high antioxidant activity, alleviation of insulin resistance and obesity, and anti-atherogenic effects (Chen et al., 2025; Peres et al., 2023).

Understanding of the spatial distribution of nutrients and bioactives across primary milling fractions is a fundamental prerequisite for the targeted, value-added utilization of whole grains in functional food design. According to the AACC International definition, a whole grain consists of the starchy endosperm, germ, and bran in their original proportions, which is crucial for preserving its nutrient density. Commercial milling processes, however, separate the grain into distinct fractions, each with unique health benefits and compositional profiles. Typically, the bran fraction is rich in dietary fiber, minerals, and antioxidant compounds (Chen et al., 2025; Huang & Lai, 2016), while the milled endosperm is predominantly starchy (Fu et al., 2025; Tullio et al., 2021). However, the distribution of nutrients across the milling fractions of wild rice (particularly the commercially relevant species of *Zizania palustris*) remains inadequately understood.

Current research, particularly on the primary cultivated species (*Zizania palustris* and *Zizania aquatica*) still faces several limitations: (i) Most studies focus on agronomic traits or whole grain properties, lacking a comparative analysis of the nutritional and functional profiles among its primary milling fractions. (ii) There is an absence of an integrated analytical framework that correlates morphological characteristics with the distribution of nutrients and bioactive activities among these fractions. (iii) Multi-fraction comparative studies with conventional rice controls are limited, making it difficult to objectively quantify the relative advantages of wild rice for specific food applications. Therefore, these limitations hinder a clear understanding of the distribution of nutrients and bioactive compounds in different fractions of wild rice, thereby constraining the targeted development of its functional food products.

Therefore, this study aims to: (i) systematically and comparatively analyze the morphological, nutritional compounds, and antioxidant activity of the whole grain, milled grain, and bran fractions of wild rice (*Zizania palustris*); (ii) compare these profiles with those of conventional white and brown rice controls; and (iii) quantify the distribution of key nutrients and bioactives across fractions. This work objectively highlights the nutritional and functional advantages of wild rice, and provides a scientific basis for its targeted application in the development of value-added functional foods.

## 2 Materials and methods

### 2.1 Materials and sample preparation

Three varieties of wild rice (*Zizania palustris*, ZP), commercially packaged by Liuhaogucang Food Technology Co., Ltd. (Guangzhou, China) and originating from the Ice Lake region of Lake Superior, Canada, were procured from local markets in China. The wild rice samples were classified into three commercial grades based on kernel length: grade No. 1 (long, >15 mm, referred to as ZP^1^), grade No. 2 (medium, 10–15 mm, ZP^2^), and grade No. 3 (short, <10 mm, ZP^3^). These three length-based grades, representing the common commercial classification for wild rice, were treated as independent samples for comparative analysis. Commercially available brown rice (BR, japonica rice) and white rice (WR, japonica rice) of the Dao Hua Xiang variety (harvested in 2025, Wuchang, Heilongjiang, China) were used as controls.

To obtain commercially relevant milled grains (MG) and bran powder (BP), whole grain kernels of wild rice and brown rice were milled using a laboratory mill (model JM3010, Mixian Technology Co., Ltd., Shenzhen, China). This equipment employs a blade grinding and rotating disc system that applies continuous shear to remove bran uniformly while minimizing high-impact fragmentation. Based on preliminary trials optimizing bran separation with minimal kernel fragmentation, a 100 g sample was milled using a laboratory mill operated at 4000 rpm and 650 W, with an applied pressure of 5 N (corresponding to the fourth gear setting) for a milling duration of 3 min. Subsequently, all samples, including the commercial WR, were ground into a fine powder using a Cyclone sample mill (UDY Corporation, Fort Collins, CO, USA) and passed through a 100-mesh sieve to obtain the grain powder. All samples were stored in airtight containers at 4 °C in the dark until analysis. A detailed sample nomenclature is provided in Supplementary Material Table S1.

### 2.2 Chemicals and reagents

All solvents and reagents were of analytical grade and purchased from Yuanye Bio-Technology Co., Ltd. (Shanghai, China). The reagents used included Folin-Ciocalteu reagent, gallic acid, rutin trihydrate, trolox, sodium carbonate, potassium acetate, aluminum chloride hexahydrate, hydrochloric acid, sulfuric acid, sodium hydroxide, nitric acid, perchloric acid, methanol, ethanol, potassium peroxomonosulfate, 2,4,6-tripyridyl-*s*-triazine (TPTZ), FeCl_3_·6H_2_O (ferric chloride), 2,2′-azino-bis (3-ethylbenzothiazoline-6-sulfonic acid) diammonium salt (ABTS), and 1,1-diphenyl-2-picrylhydrazyl (DPPH).

### 2.3 Appearance quality

The length, width, chalkiness, thousand-kernel weight, head rice ratio, and broken rice ratio of whole and milled grains were measured using a rice appearance quality analyzer (SC-E, Wanshen Test Technology Co., Hangzhou, China) according to a published method (Hu et al., 2024). Overall morphology and structural features of samples were photographed using a digital single-lens reflex camera (EOS 50D, Canon Inc., Tokyo, Japan). Milling quality was determined based on the bran yield. Specifically, a 100 g sample of whole grain was milled for 3 min using the laboratory mill described in Section 2.1. Bran yield was then calculated as the weight percentage of the collected bran relative to the initial sample weight. All measurements were performed in six replicates.

### 2.4 Morphological characteristics

The morphological characteristics of powdered whole grain (WG), milled grain (MG), and bran powder (BP) were observed using a polarizing light microscope (BA3, 10Pol, Motic Industrial Group Co., Ltd., Fujian, China) according to a reported procedure (Zhang et al., 2023). Powdered samples were dispersed on a glass slide, covered with a coverslip, and imaged at 40 × magnification.

### 2.5 Scanning electron microscopy (SEM)

The surface microstructure and cross-sectional morphology of all grain samples were observed using an Emission Scanning Electron Microscope (GeminiSEM 300, Carl Zeiss, Jena, Germany) at an accelerating voltage of 5.0 kV. The sample was attached to the sample stage, and sputter-coated with a thin layer of gold prior to observation.

### 2.6 Chemical composition

Moisture (AOAC 925.10), ash (AOAC 923.03), crude protein (Kjeldahl method, AOAC 978.04; nitrogen conversion factor: 6.25), and crude fat (Soxhlet extraction, AOAC 920.39) contents were determined according to AOAC International methods (AOAC, 2016). Total starch and amylose contents were analyzed using an enzymatic kit (K-TSTA, Megazyme, Ireland) based on AOAC Method 996.11 and the iodine-binding method (ISO 6647-2:2020), respectively. Crude fiber content was quantified by the sequential acid and alkaline digestion protocol (AOAC 930.10). All analyses were performed in triplicate, and results are expressed as grams per 100 g on a dry weight basis.

### 2.7 Total amino acid profile

Total amino acid composition was determined by an acid hydrolysis method described by Huang et al. (2014) with minor modifications. Briefly, approximately 100 mg of the sample was hydrolyzed with 10 mL of 6 M HCl in a sealed hydrolysis tube. The tube was heated in a drying oven (100 °C, 24 h) to ensure complete hydrolysis. After cooling, the hydrolysate was filtered, and a 1 mL aliquot was transferred to a glass tube and dried under reduced pressure with a Termovap concentrator. The dried residue was redissolved in 1 mL of 0.02 M HCl and filtered through a 0.22 μm membrane. The amino acid composition of the filtered samples was analyzed using a high-performance amino acid analyzer (L-8900, Hitachi, Tokyo, Japan). All measurements were performed in triplicate.

### 2.8 Mineral composition

The mineral composition was determined according to the method described by Jākobsone et al. (2018) with minor modifications. Briefly, approximately 5 g of sample was dry-ashed in a muffle furnace at 600 °C to constant weight. The ash was dissolved in 5 mL of 0.1 M HNO_3_ by heating on a hotplate at 90 °C until the solution was evaporated to near dryness. The residue was then redissolved in 10 mL of 0.1 M HNO_3_, quantitatively transferred to a 25 mL volumetric flask, and diluted to volume with distilled water. The mineral concentrations in the resulting solution were quantified using an ICP-OES instrument (iCAP 7400, Thermo Fisher Scientific, USA) with external standard calibration. All measurements were performed in triplicate.

### 2.9 Fatty acid profile

Fatty acids were extracted using a chloroform-methanol mixture (1:2, *v*/v) according to the method described by Concepcion et al. (2018). The extracts were methyl-esterified with 1.0 M sulfuric acid in methanol at 80 °C for 2 h. Analysis was performed using a gas chromatograph (Agilent 7820 GC) equipped with a flameionization detector and a capillary column (Agilent CP-Sil 88, 100 m × 0.25 mm, 0.25 μm). Helium carrier gas flow was 1.0 mL/min. The oven program was: 100 °C (hold 5 min), ramp to 240 °C at 4 °C/min, hold 15 min. Injector and detector temperatures were 250 °C and 260 °C, respectively. Peaks were identified by comparing retention times with authentic standards (Supelco 37 Component FAME Mix), and quantified using heptadecanoic acid (C17:0) methyl ester as an internal standard. All measurements were performed in triplicate.

### 2.10 Extraction of total phenolics and flavonoids

Total phenolic and flavonoid contents were extracted following the procedure described by Qiu et al. (2010) with minor modifications. Briefly, 0.50 g of sample was mixed with 20 mL of 80% (*v*/v) ethanol and ultrasonicated (power: 540 W, frequency: 40 kHz) for 30 min using an ultrasonic bath (HAMEG 8150, Germany). After centrifugation at 5000 ×*g* for 15 min, the supernatant was collected and diluted to a final volume of 100 mL with 80% ethanol.

### 2.11 Total phenolic and flavonoid contents

#### 2.11.1 Total phenolic content (TPC)

The total phenolic content (TPC) was determined using the Folin-Ciocalteu assay with modifications (Zou et al., 2011). Briefly, 5 μL of the extract sample solution (in 80% ethanol) or gallic acid standard (0.01, 0.02, 0.04, 0.06, 0.08, and 0.1 mg/mL) was mixed with 195 μL of distilled water in a well of a 96-well plate; 25 μL Folin-Ciocalteu reagent solution was then added. After 6 min, 75 μL of 7% sodium carbonate solution (Na_2_CO_3_) was added and mixed gently. The reaction mixture was kept in the dark for 60 min at room temperature, and the absorbance was measured at 765 nm using a microplate reader. The final reaction mixture volume in each well was 300 μL. The TPC was expressed as mg gallic acid equivalents per gram of dry weight sample (mg GAE/g dw) (r^2^ = 0.998). The analysis was conducted with six replicates.

#### 2.11.2 Total flavonoid content (TFC)

The total flavonoid content (TFC) was determined using the aluminum chloride colorimetric assay with modifications (Tang et al., 2015). Briefly, 50 μL of the extract sample solution (in 80% ethanol) or standard of rutin (0.1, 0.2, 0.4, 0.6, 0.8 and 1 mg/mL) was mixed with 10 μL of 5% (*w*/*v*) sodium nitrite (NaNO_2_) solution in a well of 96-well plate. After 5 min, 10 μL of 10% (w/v) aluminum chloride (AlCl_3_) was added and allowed to stand for additional 5 min, and then finally 100 μL of 0.5 M sodium hydroxide (NaOH) solution was added to the mixture and let react for another 6 min. Absorbance was measured at 510 nm. The TFC was expressed as mg of rutin equivalents per gram dry weight sample (mg RE/g dw) (r^2^ = 0.999). All determinations were performed in six replicates.

### 2.12 Antioxidant activities

#### 2.12.1 DPPH radical scavenging activity

The DPPH radical scavenging activity was determined according to the method described by Grajeda-Iglesias et al. (2016) with slight modifications. 20 μL of sample dilution in methanol and 180 μL of 150 μM DPPH radical solution were added to a 96-well plate. After incubation in the dark at 25 °C for 10 min, the absorbance was measured at 515 nm using a microplate reader. Trolox was used as a standard, and the results were expressed as Trolox equivalents (μmol TE/g). All measurements were performed in six replicates.

#### 2.12.2 ABTS radical scavenging activity

The ABTS radical scavenging activity was determined following the procedure modified by Pavlić et al. (2022) and Madrazo and Rubi (2024). The ABTS radical cation (ABTS•+) stock solution was produced by reacting 7 mM ABTS solution with 2.45 mM potassium persulfate (final concentration) and allowing the mixture to stand in the dark at room temperature for 16 h before use. This stock solution was diluted with ethanol to an absorbance of 0.7 ± 0.03 at 734 nm to obtain the working solution. For the assay, 20 μL of sample or Trolox standard was mixed with 190 μL of the ABTS• + working solution in a 96-well plate. The mixture was incubated at 30 °C for 10 min, and the absorbance was immediately measured at 734 nm. Trolox was used as the reference standard, and the results were expressed as μmol TE/g. All experiments were performed in six replicates.

#### 2.12.3 Ferric reducing antioxidant power

The FRAP activity was determined following the method of Zou et al. (2011). Briefly, the FRAP reagent was prepared fresh by mixing 10 volumes of 250 mM acetate buffer (pH 3.6), with 1 volume of 10 mM TPTZ solution in 40 mM HCl, and with 1 volume of 20 mM FeCl₃·6H₂O solution. Then, 10 μL of properly diluted samples and 30 μL of distilled water was added to 260 μL of freshly prepared FRAP reagent in a well of a 96-well plate. The plate was incubated at 37 °C throughout the reaction. After 8 min, the absorbance was measured at 593 nm. The results were expressed as micromolar trolox equivalents (TE) per gram of sample (μmol TE/g). All measurements were performed in six replicates.

### 2.13 Statistical analysis

Data are presented as mean ± standard deviation. One-way analysis of variance (ANOVA) followed by Duncan's multiple range test (*P* < 0.05) was performed using SPSS version 25 (IBM Corp., Chicago, IL, USA). Figures were plotted using Origin 8.05 (OriginLab Corp., Northampton, MA, USA).

## 3 Results and discussion

### 3.1 Appearance characteristics and milling quality of grains

**Fig. 1:**
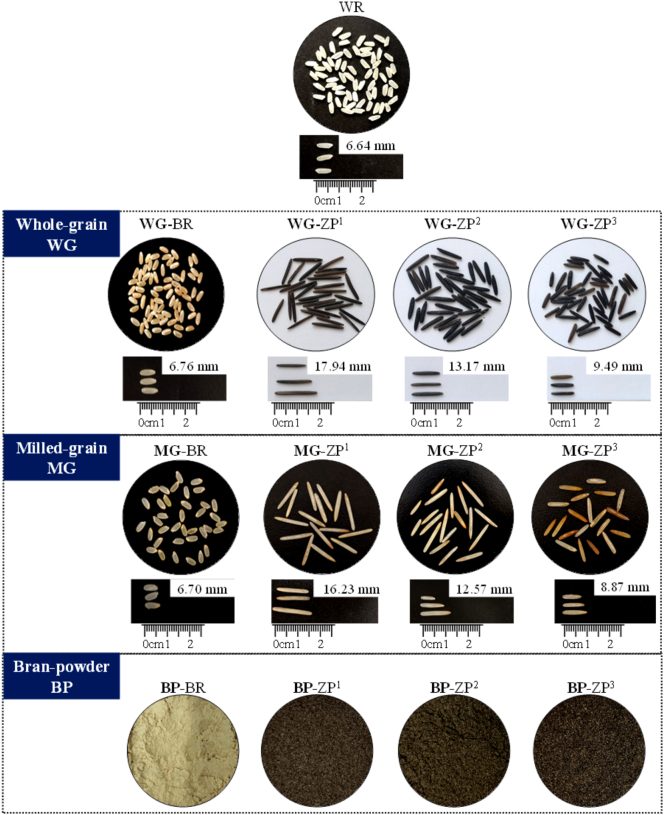
Appearance characteristics of whole grain (WG), milled grain (MG), and bran powder (BP) fractions from white rice (WR), brown rice (BR), and wild rice (*Zizania palustris*) cultivars (ZP^1^, ZP^2^, and ZP^3^). The wild rice cultivars (ZP^1^, ZP^2^, and ZP^3^) are classified by kernel length: long (> 15 mm), medium (10–15 mm), and short (< 10 mm), respectively. (For interpretation of the references to colour in this figure legend, the reader is referred to the web version of this article.)

The morphological characteristics and milling quality of the grains are summarized in Fig. 1 and Table 1. As shown in Fig. 1, white rice (WR) appeared as white, elliptical kernels, while brown rice (BR) presented a light-brown colour with a similar elliptical shape. In contrast, all three cultivars of wild rice (*Zizania palustris*, ZP) were characterized by an elongated kernel shape with acuminate ends and a glossy, black-brown pericarp, presenting a distinct morphology from the conventional rice controls (WR and BR).

**Table 1:** Appearance quality of white rice (WR), brown rice (BR) and wild rice (*Zizania palustris*, ZP^1^, ZP^2^, ZP^3^) fractions.

Sample	Grain	Uniformity length (mm)	Uniformity width (mm)	Thousand kernel weight (g)	Broken rice ratio (%)	Head rice ratio (%)	Bran weight (g/100 kernel)	Bran rice yield (%)	Chalkiness (%)
	WR	6.64 ± 0.64^d^	2.49 ± 0.62^a^	13.90 ± 0.11^h^	12.49 ± 0.97^d^	87.51 ± 0.51^a^	ND	ND	4.33 ± 0.45^a^
Whole-grain (WG)	BR	6.76 ± 0.32^d^	2.65 ± 0.19^a^	21.41 ± 0.16^g^	12.38 ± 0.90^d^	87.62 ± 1.02^a^	0.74 ± 0.01^d^	10.26 ± 0.19^a^	2.79 ± 0.71^b^
	ZP^1^	17.94 ± 1.53^a^	2.59 ± 0.11^a^	50.81 ± 0.17^a^	14.10 ± 0.36^c^	85.90 ± 0.63^a^	2.24 ± 0.02^a^	3.97 ± 0.18^b^	0.36 ± 0.19^d^
	ZP^2^	13.17 ± 0.97^b^	2.57 ± 0.24^a^	42.12 ± 0.21^c^	14.17 ± 0.43^c^	85.83 ± 0.54^a^	1.77 ± 0.01^b^	3.44 ± 0.21^c^	0.46 ± 0.05^d^
	ZP^3^	9.49 ± 1.63^c^	2.53 ± 0.39^a^	30.41 ± 0.14^e^	14.33 ± 0.44^bc^	85.67 ± 2.05^a^	1.10 ± 0.03^c^	3.00 ± 0.03^d^	0.68 ± 0.22^d^
Milled-grain (MG)	BR	6.70 ± 0.39^d^	2.60 ± 0.20^a^	14.05 ± 0.13^h^	15.49 ± 1.10^b^	84.51 ± 0.98^a^	ND	ND	4.40 ± 0.57^a^
	ZP^1^	16.23 ± 1.82^a^	2.54 ± 0.29^a^	48.41 ± 0.19^b^	17.07 ± 0.90^a^	82.93 ± 1.02^a^	ND	ND	1.07 ± 0.52^d^
	ZP^2^	12.57 ± 1.90^b^	2.53 ± 0.24^a^	34.42 ± 0.25^d^	17.17 ± 0.36^a^	82.83 ± 0.63^a^	ND	ND	1.93 ± 0.59^c^
	ZP^3^	8.87 ± 1.05^cd^	2.50 ± 0.27^a^	26.50 ± 0.11^f^	17.19 ± 0.43^a^	82.81 ± 0.54^a^	ND	ND	1.93 ± 0.43^c^

Data are represented as the mean ± standard deviation (n = 6). Different lowercase superscripts (a-h) in the same column indicate significant differences among samples (*P* < 0.05). The grain fractions include whole grain (WG) and milled grain (MG). ZP^1^, ZP^2^, and ZP^3^ indicate different cultivars of wild rice (*Zizania palustris*, ZP), classified by kernel length as long (> 15 mm), medium (10–15 mm), and short (< 10 mm), respectively. ND: not detected.

A notable visual difference was observed in the colour of the milling fractions (Fig. 1). The milled grains of ZP (MG-ZP) exhibited a distinct yellow and translucent appearance, particularly those of ZP^3^, which contrasted sharply with the opaque white of milled brown rice (MG-BR). Correspondingly, ZP bran was dark brown compared to the yellowish-brown BR bran, suggesting a higher concentration of pigmented, likely phenolic, compounds in the outer layers of wild rice. This intense pigmentation suggests a potentially higher accumulation of phenolic and flavonoid compounds in ZP, because the pigments of cereal bran are commonly associated with their high antioxidant activities (Huang & Lai, 2016). These observations provide preliminary visual evidence for a distinct phytochemical profile in ZP, which is quantified in Sections 3.7 and 3.8.

As shown in Table 1, the average length, width and thousand-kernel weight of ZP were approximately 13.50 mm, 2.60 mm, and 41.20 g, respectively, which were markedly more slender and heavier than those of WR (6.64 mm, 2.49 mm, 13.90 g) and BR (6.76 mm, 2.65 mm, 21.41 g). Among the ZP cultivars, ZP^1^ displayed the highest values for both length (17.94 mm) and thousand-kernel weight (50.81 g), followed by ZP^2^ and ZP^3^. Notably, ZP showed significantly lower chalkiness compared to both WR and BR. Since lower chalkiness is highly associated with superior grain appearance (Shi et al., 2021), this trait suggests an advantage for ZP in visual and market acceptability. However, ZP showed a slightly lower head rice ratio and a higher broken rice ratio than BR after milling, likely attributable to its longer grain kernel, which is more susceptible to breakage during milling.

Regarding bran yield (weight percentage of bran to whole grain), ZP cultivars were lower than that of BR. However, due to its significantly larger kernel size and higher thousand-kernel weight, the absolute bran weight obtainable per 100 kernels of ZP (ranging from 1.10 to 2.24 g) was substantially higher, approximately 1.5–3.0 times that of BR (0.74 g). For bran production, this distinction between bran yield (percentage) and absolute bran weight is important for evaluating bran production potential. This difference is attributed to the significantly larger kernel size and higher thousand-kernel weight of ZP. Consequently, despite a lower proportional bran yield in ZP than BR, the actual quantity of bran obtainable from ZP is markedly higher.

Overall, ZP is morphologically and qualitatively distinct from conventional rice, characterized by longer, heavier kernels, lower chalkiness, and a darker bran layer. The milled ZP exhibited a distinct yellow and translucent appearance, while the milled brown rice was white. Among the three ZP cultivars, ZP^1^ had the longest and heaviest grains, whereas ZP^3^ yielded the darkest bran powder.

### 3.2 Microstructural and morphological characteristics of grain fractions

**Fig. 2:**
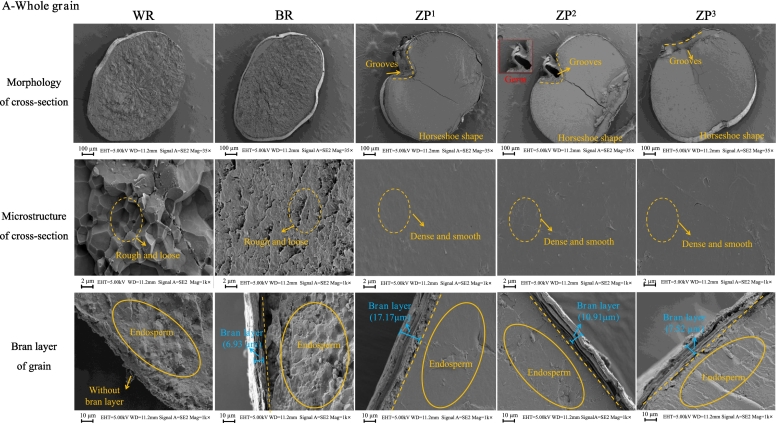

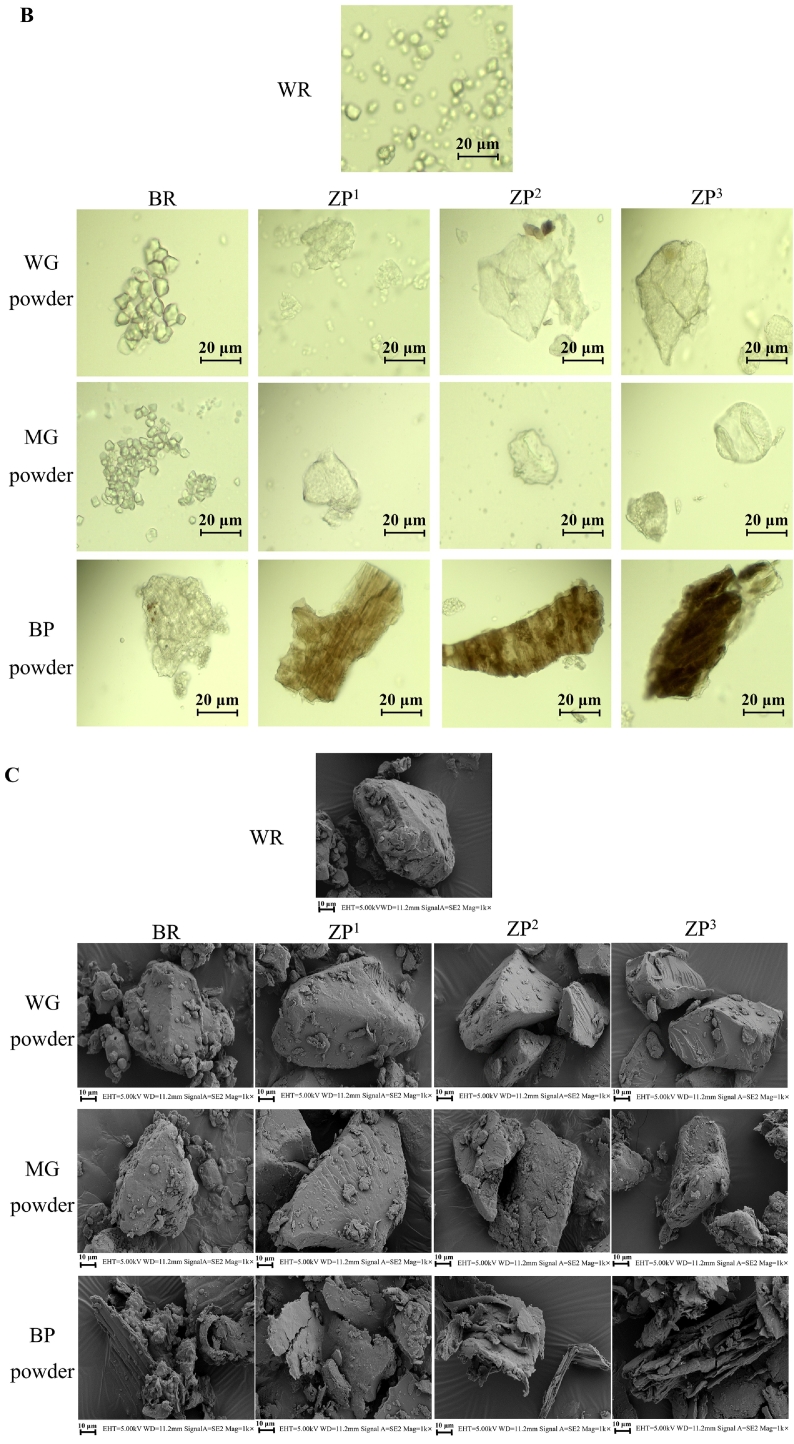
Microstructural and morphological characteristics of whole grain (WG), milled grain (MG), and bran powder (BP) fractions from white rice (WR), brown rice (BR), and wild rice (*Zizania palustris*) cultivars (ZP^1^, ZP^2^, ZP^3^). (A) Scanning electron microscopy (SEM) images of the cross-section, surface microstructure, and bran layer thickness of whole grains. (B) Light microscopy images of powdered whole grain (WG), milled grain (MG), and bran powder (BP). (C) SEM images of the granular morphology of powdered WG, MG, and BP. The wild rice cultivars (ZP^1^, ZP^2^, and ZP^3^) are classified by kernel length: long(> 15 mm), medium (10–15 mm), and short (< 10 mm), respectively. (For interpretation of the references to colour in this figure legend, the reader is referred to the web version of this article.)

The microstructural and morphological features of whole grain (WG), milled grain (MG), and bran powder (BP) fractions derived from WR, BR, and ZP are presented in Fig. 2. Cross-sectional views of the whole grains (Fig. 2A) showed obvious differences in endosperm organization among the samples. The WR endosperm exhibited a honeycomb-like structure with a rough surface, primarily composed of polyhedral and angular starch granules, consistent with previous reports (Zhu et al., 2023). BR showed a similar endosperm structure, which was enclosed by a bran layer, and the matrix exhibited a less compact structure.

In contrast, the ZP endosperm displayed an exceptionally dense and smooth internal fracture surface with minimal pores, compared with WR and BR. This compact microstructure suggests a strong, continuous matrix, likely protein-rich, surrounding the starch granules, which is a structural feature associated with higher protein content in cereals (Wu et al., 2025). This interpretation is further substantiated by the quantitative protein analysis presented in Section 3.3, where ZP exhibited significantly higher protein content than both WR and BR. Interestingly, as shown in Fig. 2A, ZP grains showed a distinct horseshoe-shaped morphology in cross-section (most evident in ZP^1^ due to its longer kernel length), attributable to a deep longitudinal groove in the germinal region. This distinctive morphology, characteristic of the Zizania genus and potentially an adaptive trait related to its aquatic habitat, may enhance nutrient storage capacity as observed in other aquatic cereals (Yang et al., 2025; Yu et al., 2020).

The granular morphologies of the powdered fractions are shown in Fig. 2B and C. Powders from whole and milled grains of WR and BR exhibited typical polyhedral structures, whereas ZP powders appeared more angular and irregular. All bran fractions showed irregular and stacked lamellar structures. However, ZP bran was markedly darker (blackish-brown) and significantly thicker (17.17, 10.91, and 7.52 μm for ZP^1^, ZP^2^, and ZP^3^, respectively) than BR bran (6.93 μm). The bran thickness and coloration are recognized as genetically influenced traits with direct implications for nutritional quality (Chen et al., 2025). The greater thickness and intense pigmentation of ZP bran provide a morphological basis for its higher content of bioactive compounds, such as phenolics and flavonoids, relative to BR and WR (Sections 3.3 and 3.7).

Overall, these unique microstructural findings showed that ZP has a dense endosperm, a thicker, more heavily pigmented bran layer, and a distinctive horseshoe-shaped cross-section compared to conventional rice. These structural characteristics provide a basis for understanding its unique textural properties, cooking performance, and enhanced nutritional potential, particularly in terms of dietary fiber and phytochemical content.

### 3.3 Chemical composition of grain fractions

The chemical composition of WG, MG, and BP fractions from WR, BR, and ZP is presented in Table 2. Significant differences (*P* < 0.05) were observed between ZP and conventional rice across all fractions.

**Table 2:** Nutritional composition of white rice (WR), brown rice (BR) and three kinds of wild rice (*Zizania palustris*, ZP^1^, ZP^2^, ZP^3^) fractions.

Sample	Grain	Moisture content (g/100 g)	Ash content (g/100 g)	Total starch content (g/100 g)	Amylose starch Content (g/100 g)	Amylopectin Content (g/100 g)	Proteins content (g/100 g)	Fat content (g/100 g)	Crude fiber content (g/100 g)
	WR	10.29 ± 0.22^fg^	0.23 ± 0.04^e^	81.83 ± 0.03^a^	16.00 ± 0.14^i^	65.83 ± 0.15^a^	6.98 ± 0.16^k^	0.42 ± 0.02^i^	0.25 ± 0.08^k^
Whole-grain (WG)	BR	12.32 ± 0.31^d^	0.38 ± 0.17^e^	73.06 ± 0.21^d^	16.59 ± 0.11^h^	56.47 ± 0.08^c^	8.49 ± 0.13^j^	2.94 ± 0.16^e^	2.81 ± 0.11^h^
	ZP^1^	9.50 ± 0.11^h^	0.82 ± 0.08^d^	64.71 ± 0.09^e^	29.66 ± 0.09^d^	35.05 ± 0.11^i^	13.00 ± 0.17^e^	0.71 ± 0.08^gh^	11.26 ± 0.16^e^
	ZP^2^	10.37 ± 0.15^f^	0.91 ± 0.07^d^	63.63 ± 0.33^f^	27.48 ± 0.07^e^	36.15 ± 0.13^h^	13.18 ± 0.12^e^	0.93 ± 0.10^fg^	10.98 ± 0.09^f^
	ZP^3^	10.93 ± 0.20^e^	1.20 ± 0.09^c^	63.34 ± 0.22^g^	26.09 ± 0.21^f^	37.25 ± 0.10^g^	13.67 ± 0.15^d^	0.98 ± 0.12^f^	9.88 ± 0.07^g^
Milled-grain (MG)	BR	10.04 ± 0.12^g^	0.24 ± 0.12^e^	81.94 ± 0.13^a^	18.39 ± 0.12^g^	63.55 ± 0.22^b^	7.03 ± 0.06^k^	0.41 ± 0.05^i^	0.34 ± 0.12^k^
	ZP^1^	8.99 ± 0.13^i^	0.59 ± 0.17^d^	80.27 ± 0.22^b^	36.78 ± 0.06^a^	43.49 ± 0.17^f^	8.69 ± 0.09^i^	0.59 ± 0.07^hi^	0.90 ± 0.08^i^
	ZP^2^	8.79 ± 0.14^ij^	0.63 ± 0.20^d^	79.52 ± 0.08^c^	34.35 ± 0.07^b^	45.17 ± 0.09^e^	9.59 ± 0.10^h^	0.73 ± 0.09^fgh^	0.74 ± 0.20^ij^
	ZP^3^	8.58 ± 0.13^j^	0.76 ± 0.19^d^	79.28 ± 0.04^c^	32.66 ± 0.15^c^	46.62 ± 0.20^d^	9.99 ± 0.13^g^	0.79 ± 0.13^fgh^	0.60 ± 0.09^j^
Bran-powder (BP)	BR	18.39 ± 0.09^a^	2.00 ± 0.12^b^	23.64 ± 0.13^h^	ND	ND	12.77 ± 0.14^f^	10.30 ± 0.11^a^	32.90 ± 0.12^d^
	ZP^1^	15.87 ± 0.14^c^	2.95 ± 0.13^a^	13.58 ± 0.20^k^	ND	ND	18.51 ± 0.09^c^	3.80 ± 0.16^d^	45.29 ± 0.11^a^
	ZP^2^	16.68 ± 0.10^b^	3.01 ± 0.10^a^	17.51 ± 0.14^j^	ND	ND	18.78 ± 0.06^b^	4.07 ± 0.24^c^	39.95 ± 0.08^b^
	ZP^3^	16.84 ± 0.08^b^	3.13 ± 0.08^a^	18.47 ± 0.11^i^	ND	ND	19.31 ± 0.11^a^	4.89 ± 0.12^b^	37.36 ± 0.19^c^

Data are represented as the mean ± standard deviation (*n* = 3). Different lowercase superscripts (a-k) in the same column indicate significant differences among samples (*P* < 0.05). The grain fractions include whole grain (WG), milled grain (MG), and bran powder (BP). ZP^1^, ZP^2^, and ZP^3^ indicate different cultivars of wild rice (*Zizania palustris*, ZP), classified by kernel length as long (> 15 mm), medium (10–15 mm), and short (< 10 mm), respectively. ND: not detected.

In the whole grain fractions (WG), ZP exhibited a nutritionally distinct profile characterized by substantially higher contents of protein, ash, and crude fiber, but lower total starch and fat compared to WR and BR (Table 2). Specifically, the average crude fiber content in ZP (∼10.70 g/100 g) was approximately 3.8 to 42.8 times higher than that of BR (2.81 g/100 g) and WR (0.25  g/100 g), respectively, consistent with previous reports (Yan et al., 2018; Mohidem et al., 2022). This dramatic difference is consistent with the observation of a notably thicker bran layer in ZP (Fig. 2A, C), as dietary fiber is predominantly located in the outer layers of the grain. Similarly, the average protein content of ZP (∼13.30 g/100 g) was approximately 1.6- and 1.9-fold higher than that of BR (8.49 g/100 g) and WR (6.98 g/100 g), respectively, and also surpassed that of pigmented whole grains such as black rice (Bartwal et al., 2020). These values are consistent with those reported for North American wild rice populations (Surendiran et al., 2014; Yang et al., 2025) and are considerably higher than those found in other pigmented rice varieties, including black and red rice (Melini & Acquistucci, 2017). This positions ZP with the highest protein content among commonly consumed whole grains, corroborating the inference from the dense endosperm microstructure (Fig. 2A) (Devkota et al., 2025). Additionally, ZP showed a markedly lower total starch content (∼63.90 g/100 g) compared to WR (81.83 g/100 g) and BR (73.06 g/100 g), which is consistent with the findings of Surendiran et al. (2014). The unique combination of high protein and fiber with lower starch content suggests a potential for slower carbohydrate digestion, which is consistent with previous reports (Javanmardi et al., 2021; Kumar et al., 2022). Among ZP cultivars, ZP^1^ contained the lowest amylopectin and the highest amylose contents, while ZP^3^ exhibited the highest protein content. All ZP cultivars had lower moisture and fat contents than BR, which may reduce the risk of lipid oxidation during storage or processing (Fu et al., 2025).

The milling process significantly altered the nutritional profiles of grains. Compared to the whole grains, the milled grains exhibited a decrease in moisture, ash, and protein, while total starch increased due to the removal of the bran. Notably, the protein content in milled ZP (MG-ZP, 8.69–9.99 g/100 g) remained significantly higher than in milled BR (7.03 g/100 g), indicating that the inherent nutritional advantage of ZP is partially preserved even after milling. For the bran fraction, ZP bran contained the highest levels of dietary fiber (45.29 g/100 g in ZP^1^) and protein content (19.31 g/100 g in ZP^3^) among all bran samples. This indicates that the darker, thicker bran of ZP (Fig. 2A, C) is not merely a morphological trait but correlates with a superior density of key nutrients.

Overall, ZP has a unique and nutritionally advantageous chemical composition compared to conventional rice, characterized by higher protein, ash, and dietary fiber, and lower starch and fat content. These benefits are most concentrated in the bran but are also notable in the whole grain. Among ZP cultivars, ZP^1^ had the highest dietary fiber content, whereas ZP^3^ exhibited the highest protein, starch, and fat contents.

### 3.4 Amino acids profile

**Fig. 3:**
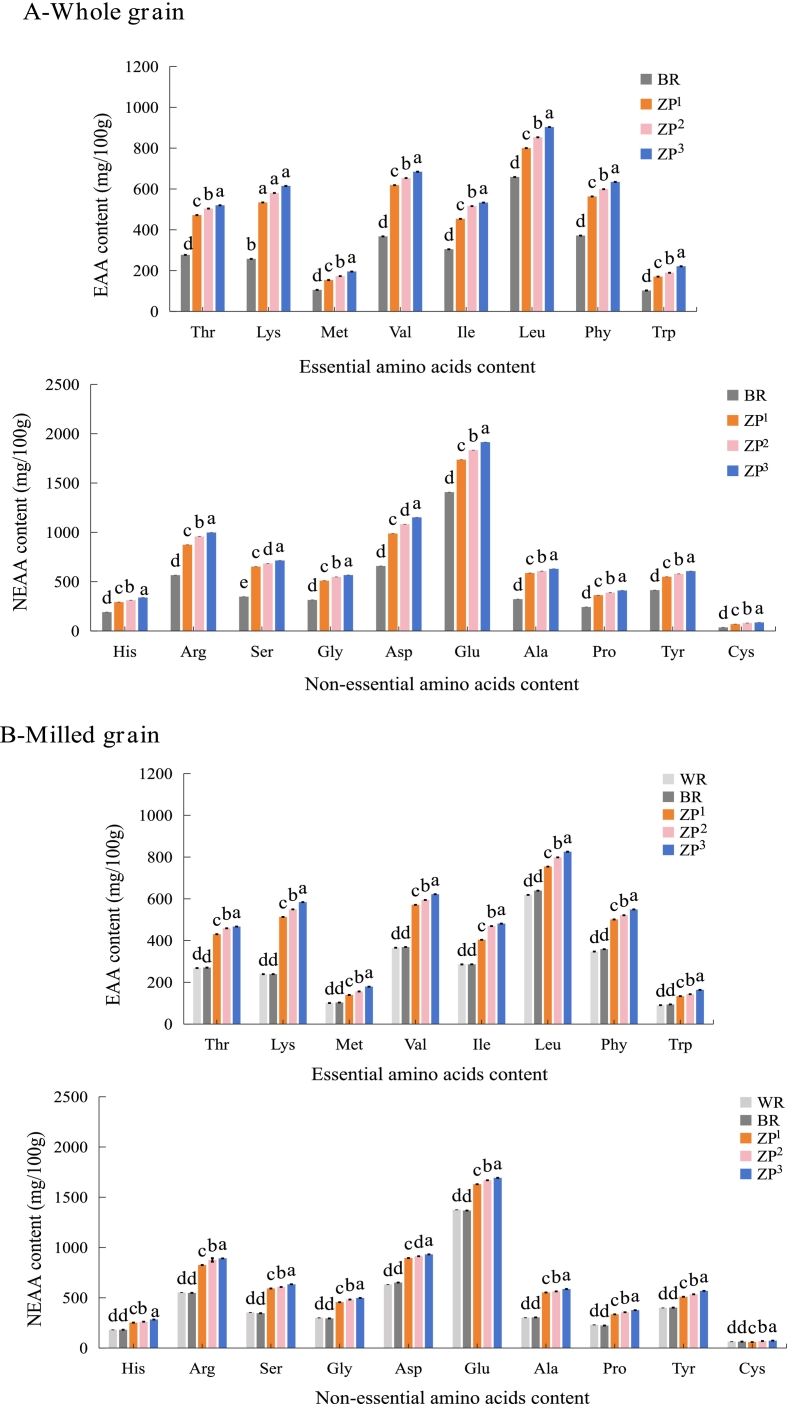

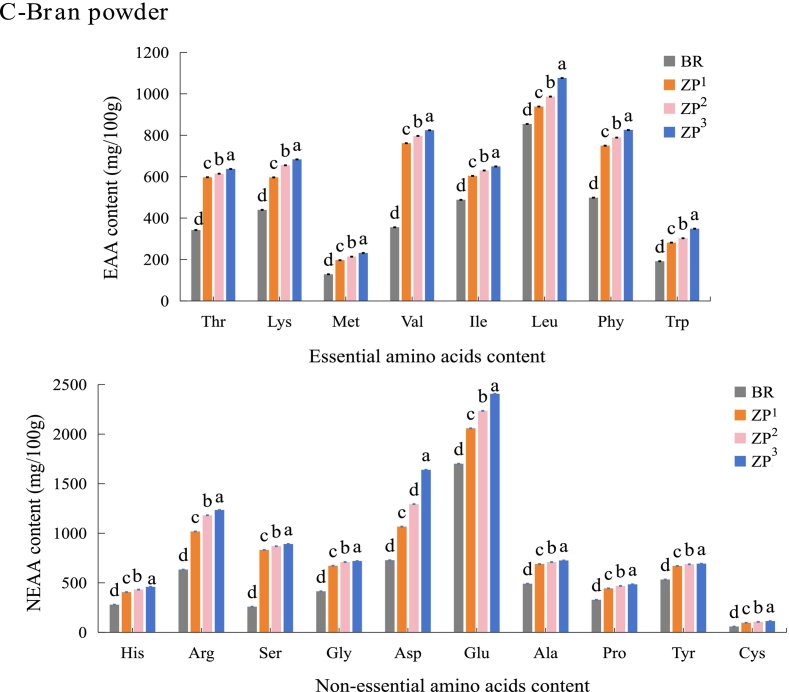
Amino acid content of whole grain (WG), milled grain (MG), and bran (BP) fractions from white rice (WR), brown rice (BR), and wild rice (*Zizania palustris*) cultivars (ZP^1^, ZP^2^, ZP^3^). (A-C) illustrate the essential (EAA) and non-essential (NEAA) amino acid contents of WG, MG, and BP, respectively. The amino acid abbreviations are as follows: Threonine (Thr), Lysine (Lys), Methionine (Met), Valine (Val), Isoleucine (Ile), Leucine (Leu), Phenylalanine (Phe), Tryptophan (Trp), Histidine (His), Arginine (Arg), Serine (Ser), Glycine (Gly), Aspartic acid (Asp), Glutamic acid (Glu), Alanine (Ala), Proline (Pro), Tyrosine (Tyr), and Cysteine (Cys). All determinations were performed in six independent replicates (*n* = 6). Different lowercase letters (a-e) above bars indicate significant differences among different grain samples within the same amino acid (*P* < 0.05). The wild rice cultivars (ZP^1^, ZP^2^, and ZP^3^) are classified by kernel length: long (> 15 mm), medium (10–15 mm), and short (< 10 mm), respectively. (For interpretation of the references to colour in this figure legend, the reader is referred to the web version of this article.)

Significant differences in amino acid composition were observed between wild rice (ZP) and the conventional rice controls (WR and BR) (Fig. 3). In the WG fraction, the total amino acid (TAA) content of ZP (∼11,081.00 mg/100 g) was approximately 1.6-fold higher than that of WR (6669.24 mg/100 g) and BR (6943.15 mg/100 g) (*P* < 0.05). Lysine (Lys) is widely recognized as the primary limiting amino acid in conventional rice, a well-known nutritional constraint in cereal-based diets that necessitates dietary supplementation through lysine-rich animal proteins or complementary plant sources (like legumes) (Yang et al., 2016). Notably, the Lys content in ZP (577.00 mg/100 g) was approximately 2.3-fold higher than in WR (238.92 mg/100 g) and BR (257.42 mg/100 g). This remarkably higher lysine content suggests that ZP could serve as a valuable plant-based complement to conventional cereals, potentially enhancing dietary protein quality without heavy reliance on animal-derived supplements or legume fortification. This finding is consistent with a recent published study showing that wild rice possesses a more favorable essential amino acid profile than conventional white and brown rice (Sumczynski et al., 2018). As expected, Lys was the first-limiting amino acid in WR and BR, whereas sulfur-containing amino acids (methionine + cysteine) were the first-limiting in ZP. Furthermore, the ratio of essential to total amino acids (EAA/TAA) in ZP whole grain (∼37%) was closer to the FAO/WHO ideal pattern (40%) than that of WR (34%) and BR (35%), indicating a more balanced amino acid composition (Mohammadzadeh & Feizy, 2025). In addition, no significant differences in EAA/TAA were observed among ZP cultivars.

Among non-essential amino acids (NEAAs), glutamic acid (Glu) was the most abundant in ZP (∼1828.00 mg/100 g), with levels 1.3-fold higher than those in WR and BR. As a key contributor to the umami taste, the high concentration of Glu may enhance the flavor profile of ZP-based products (Wu et al., 2024). Other predominant NEAAs included aspartate (Asp) and arginine (Arg), which were also present at significantly higher concentrations in ZP than in WR and BR. Among the ZP cultivars, ZP^3^ exhibited the highest concentrations of most NEAAs.

Milling reduced total amino acid content due to bran removal, as expected. However, several key essential amino acids, such as lysine, threonine, and branched-chain amino acids (valine, leucine, isoleucine) remained notably higher in milled ZP than in milled controls (Fig. 3B). These findings indicate that the specific amino acid profile of ZP is partially preserved after milling, suggesting that its nutritional benefits, particularly regarding amino acid balance, are preserved even in refined forms.

Amino acids were highly concentrated in the bran (BP) fraction for all grains (Fig. 3C). ZP bran contained the highest levels, with lysine (645.10 mg/100 g) and glutamic acid contents (2233.10 mg/100 g), approximately 1.3-fold higher than in BR bran, with an EAA/TAA ratio of ∼38%. Notably, even after milling, the EAA/TAA ratio of ZP (∼36%) remained higher than that of the milled controls.

Therefore, ZP exhibits a quantitatively and qualitatively enhanced amino acid profile compared to that of conventional rice, as indicated by its higher total amino acid content, higher levels of key amino acids such as Lys and Glu, and a more balanced EAA/TAA ratio. These advantages are most concentrated in the bran but remain significant in the milled grain, highlighting its potential not only as a high-quality plant protein source but specifically as a targeted ingredient for addressing lysine deficiency in cereal-based functional foods.

### 3.5 Mineral content

The mineral compositions of ZP, WR, and BR are summarized in Table 3. Compared with rice controls, ZP contained significantly higher levels of both macroelements and trace elements among all samples. For the whole grain (WG) fraction, the total mineral content of ZP (∼897.80 mg/100 g) was approximately 3.3-fold and 2.9-fold higher than that of WR (269.83 mg/100 g) and BR (310.78 mg/100 g), respectively. Among ZP cultivars, ZP^3^ exhibited the highest mineral content (1006.88 mg/100 g), followed by ZP^2^ (910.56 mg/100 g) and ZP^1^ (775.83 mg/100 g). The mineral content, such as potassium (K), phosphorus (P), and magnesium (Mg) were the predominant macroelements in all samples, which were approximately 3.0-, 3.4-, and 5.2-fold higher than those in WR and BR, respectively, which is consistent with the findings of Sumczynski et al. (2018). Additionally, the calcium (Ca) content in ZP was approximately 4.3- and 9.4-fold higher than in BR and WR, respectively.

**Table 3:** The minerals compositions of white rice (WR), brown rice (BR) and wild rice (*Zizania palustris*, ZP^1^, ZP^2^, ZP^3^) fractions.

Sample		Cumulative macroelements and trace elements content in different varieties of rice (mg/100 g)																		
		Macronutrients					Essential micronutrients						Potentially beneficial trace elements			Potentially toxic elements				Total mineral
		K	P	Mg	Ca	Na	Mn	Zn	Fe	Cu	Cr	Co	B	Ni	V	As	Li	Al	Pb	
	WR	117.11 ± 1.15^j^	95.11 ± 1.13^l^	51.18 ± 1.14^j^	4.19 ± 0.02^j^	0.57 ± 0.12^g^	0.64 ± 0.09^g^	0.45 ± 0.01^g^	0.38 ± 0.01^i^	0.16 ± 0.01^f^	0.08 ± 0.01^gh^	0.03 ± 0.00^g^	0.02 ± 0.01^j^	0.03 ± 0.00^h^	ND	0.02 ± 0.00^g^	0.01 ± 0.00^f^	ND	ND	264.83 ± 1.21^m^
Whole-grain (WG)	BR	123.31 ± 1.11^i^	98.29 ± 2.12^k^	78.19 ± 0.21^h^	7.10 ± 0.12^h^	0.78 ± 0.21^g^	1.01 ± 0.15^f^	0.61 ± 0.02^g^	0.51 ± 0.01^gh^	0.22 ± 0.01^f^	0.08 ± 0.01^gh^	0.03 ± 0.00^g^	0.18 ± 0.01^h^	0.17 ± 0.00^g^	0.02 ± 0.00^g^	0.02 ± 0.00^g^	0.01 ± 0.00^f^	ND	ND	310.78 ± 1.59^k^
	ZP^1^	286.86 ± 1.16^f^	251.91 ± 1.11^g^	203.97 ± 0.24^g^	22.74 ± 0.11^g^	2.35 ± 0.21^f^	2.56 ± 0.16^e^	2.01 ± 0.11^f^	1.87 ± 0.14^f^	0.45 ± 0.01^d^	0.19 ± 0.01^e^	0.12 ± 0.02^f^	0.33 ± 0.01^g^	0.33 ± 0.01^f^	0.06 ± 0.00^e^	0.07 ± 0.00^e^	0.03 ± 0.00^d^	ND	ND	775.83 ± 1.24^f^
	ZP^2^	332.80 ± 1.12^e^	292.19 ± 1.11^e^	239.72 ± 2.12^e^	32.78 ± 0.11^f^	2.88 ± 0.21^e^	3.10 ± 0.23^d^	2.48 ± 0.12^d^	2.23 ± 0.13^d^	0.57 ± 0.01^c^	0.25 ± 0.01^c^	0.13 ± 0.01^ef^	0.44 ± 0.01^f^	0.44 ± 0.01^e^	0.12 ± 0.01^c^	0.08 ± 0.00^d^	0.05 ± 0.01^c^	ND	ND	910.56 ± 1.63^e^
	ZP^3^	364.58 ± 1.14^c^	322.01 ± 2.11^d^	266.56 ± 2.16^d^	39.40 ± 0.41^e^	3.34 ± 0.23^d^	3.44 ± 0.13^c^	2.77 ± 0.14^c^	2.45 ± 0.04^c^	0.63 ± 0.01^bc^	0.28 ± 0.01^b^	0.15 ± 0.01^de^	0.58 ± 0.01^e^	0.58 ± 0.01^d^	0.13 ± 0.01^bc^	0.09 ± 0.00^c^	0.06 ± 0.00^b^	ND	ND	1006.88 ± 2.18^d^
Milled-grain (MG)	BR	118.53 ± 0.48^j^	97.97 ± 0.13^k^	66.67 ± 0.15^i^	3.83 ± 0.11^j^	0.57 ± 0.11^g^	0.79 ± 0.11^fg^	0.46 ± 0.02^g^	0.43 ± 0.02^hi^	0.20 ± 0.01^f^	0.07 ± 0.01^h^	0.03 ± 0.00^g^	0.02 ± 0.00^j^	0.02 ± 0.00^h^	0.02 ± 0.00^g^	0.02 ± 0.00^g^	0.01 ± 0.00^f^	ND	ND	294.03 ± 1.05^l^
	ZP^1^	124.83 ± 0.28^i^	130.94 ± 0.17^j^	77.68 ± 0.12^h^	4.17 ± 0.14^j^	0.61 ± 0.12^g^	0.86 ± 0.12^fg^	0.55 ± 0.02^g^	0.51 ± 0.01^gh^	0.21 ± 0.01^f^	0.09 ± 0.01^fg^	0.04 ± 0.00^g^	0.03 ± 0.00^ij^	0.03 ± 0.00^h^	0.02 ± 0.01^g^	0.02 ± 0.00^g^	0.01 ± 0.00^f^	ND	ND	340.74 ± 1.36^j^
	ZP^2^	125.12 ± 0.22^i^	145.58 ± 0.22^i^	79.72 ± 0.11^h^	5.57 ± 0.15^i^	0.62 ± 0.11^g^	0.89 ± 0.12^fg^	0.57 ± 0.00^g^	0.53 ± 0.00^gh^	0.22 ± 0.01^f^	0.09 ± 0.00^fg^	0.04 ± 0.00^g^	0.04 ± 0.00^i^	0.04 ± 0.00^h^	0.04 ± 0.00^f^	0.02 ± 0.00^g^	0.02 ± 0.00^e^	ND	ND	358.95 ± 2.06^i^
	ZP^3^	130.80 ± 0.11^h^	152.88 ± 0.13^h^	79.73 ± 0.24^h^	6.53 ± 0.10^h^	0.64 ± 0.11^g^	0.91 ± 0.13^fg^	0.59 ± 0.02^g^	0.56 ± 0.00^g^	0.23 ± 0.02^f^	0.10 ± 0.00^f^	0.04 ± 0.00^g^	0.04 ± 0.00^i^	0.04 ± 0.00^h^	0.04 ± 0.00^f^	0.02 ± 0.00^g^	0.02 ± 0.00^e^	ND	ND	372.95 ± 2.07^h^
Bran-powder (BP)	BR	191.98 ± 1.08^g^	281.20 ± 1.14^f^	213.75 ± 2.08^f^	43.64 ± 0.24^d^	3.14 ± 0.02^d^	3.11 ± 0.12^d^	2.23 ± 0.11^e^	2.02 ± 0.01^e^	0.32 ± 0.01^e^	0.21 ± 0.01^d^	0.14 ± 0.01^de^	0.63 ± 0.01^d^	0.63 ± 0.01^d^	0.08 ± 0.01^d^	0.05 ± 0.00^f^	0.03 ± 0.00^d^	ND	ND	742.88 ± 1.21^g^
	ZP^1^	361.38 ± 2.04^d^	339.86 ± 2.06^c^	301.18 ± 2.03^c^	51.69 ± 0.31^c^	4.64 ± 0.01^c^	4.16 ± 0.21^b^	3.89 ± 0.11^b^	3.48 ± 0.01^b^	0.59 ± 0.01^c^	0.22 ± 0.01^d^	0.16 ± 0.01^bc^	0.84 ± 0.01^c^	0.85 ± 0.02^c^	0.12 ± 0.01^c^	0.13 ± 0.01^b^	0.05 ± 0.01^c^	ND	ND	1073.52 ± 2.68^c^
	ZP^2^	381.67 ± 1.04^b^	349.83 ± 2.10^b^	310.51 ± 2.56^b^	60.94 ± 0.71^b^	4.89 ± 0.11^b^	4.55 ± 0.24^a^	4.07 ± 0.21^b^	3.58 ± 0.01^ab^	0.68 ± 0.11^a^	0.29 ± 0.01^b^	0.17 ± 0.01^b^	0.93 ± 0.01^b^	0.96 ± 0.05^b^	0.14 ± 0.01^ab^	0.13 ± 0.01^b^	0.06 ± 0.00^b^	ND	ND	1123.81 ± 3.27^b^
	ZP^3^	390.50 ± 2.01^a^	353.57 ± 1.05^a^	337.37 ± 1.05^a^	66.78 ± 0.91^a^	5.38 ± 0.13^a^	4.77 ± 0.14^a^	4.28 ± 0.23^a^	3.66 ± 0.09^a^	0.72 ± 0.11^b^	0.32 ± 0.02^a^	0.19 ± 0.01^a^	1.15 ± 0.01^a^	1.13 ± 0.10^a^	0.15 ± 0.01^a^	0.14 ± 0.01^a^	0.07 ± 0.00^a^	ND	ND	1169.89 ± 4.16^a^

Data are represented as the mean ± standard deviation (n = 3). Different lowercase superscripts (a-m) in the same column indicate significant differences among samples (*P* < 0.05). The grain fractions include whole grain (WG), milled grain (MG), and bran powder (BP). ZP^1^, ZP^2^, and ZP^3^ indicate different cultivars of wild rice (*Zizania palustris*, ZP), classified by kernel length as long (> 15 mm), medium (10–15 mm), and short (< 10 mm), respectively. ND: not detected.

Regarding essential trace elements, the contents of manganese (Mn), zinc (Zn), and iron (Fe) were 4- to 6-fold higher in ZP than in the conventional rice controls. These values exceed those reported for many commercial rice varieties and are comparable to the richest mineral sources among pigmented cereals (Bartwal et al., 2020). It is known that Zn and Fe deficiencies are prevalent global health issues; these findings indicate the potential of ZP as a dietary source for micronutrient enhancement. Furthermore, ZP also contained higher levels of other beneficial trace elements, including boron (B), nickel (Ni), and vanadium (V). Previous studies have indicated that vanadium compounds can exhibit insulin-mimetic properties in in vitro and animal models (Kioseoglou et al., 2015). However, in the present study, V was not detected in WR, but was identified in both ZP and BR. Notably, the V content in ZP was 5.2 times higher than that in BR. Furthermore, no potentially toxic trace elements (e.g., Al, Pb) were detected in any samples, and the levels of all other monitored toxic elements were within the safety thresholds established by the FAO/WHO (Rahman & Islam, 2019).

The mineral density of ZP remained appreciable even after milling. The MG fraction of BR was 294.03 mg/100 g, whereas milled ZP maintained significantly higher mineral content (∼357.60 mg/100 g) than milled BR, with ZP^3^ containing the highest. As expected, the BP was the most mineral-dense fraction compared to WG and MG. ZP bran exhibited the highest total mineral content (1073.52–1169.89 mg/100 g), significantly higher than BR bran (742.88 mg/100 g), and was particularly rich in essential micronutrients, such as Mn, Zn, Fe, and Cu. Furthermore, potentially beneficial trace elements like B, Ni, and V were detected in both the MG and BP fractions, and no toxic elements like Al and Pb were detected.

Overall, compared to WR and BR, ZP (particularly ZP^3^) constitutes a dense source of both macro- and micro-elements. The high concentrations of nutritionally critical minerals such as Zn, Fe, and Mg, combined with the presence of potentially beneficial trace elements like V, positions ZP as a natural ingredient for the nutritional fortification of staple foods. This nutritional advantage is most concentrated in the bran fraction and remains significant even in the milled grain, indicating that ZP, and especially its bran, can be used as a valuable ingredient for dietary mineral enhancement in functional foods.

### 3.6 Fatty acid profile

The fatty acid content is a critical determinant of nutritional quality and oxidative stability in cereals. As presented in Table 4, ZP (WG fractions) exhibited a distinct fatty acid profile characterized by a lower proportion of saturated fatty acids (SFAs, ∼13%) and a correspondingly higher proportion of unsaturated fatty acids (UFAs, ∼87%) compared to WR and BR (∼21% SFAs, ∼79% UFAs). The high dietary intake of SFAs is an established risk factor for cardiovascular disease (Zhao et al., 2023). Thus, the lower SFAs content in ZP suggests a more favorable nutritional profile for health benefits. Moreover, oleic acid (C18:1n9c) and eicosenoic acid (C20:1) were the principal monounsaturated fatty acids (MUFAs) in ZP.

**Table 4:** Fatty acids composition in white rice (WR), brown rice (BR) and wild rice (*Zizania palustris*, ZP^1^, ZP^2^, ZP^3^) fractions.

Sample		Fatty acid composition (% of total fatty acids)											
		Saturated fatty acids (SFA)			Monounsaturated fatty acids (MUFA)		Polyunsaturated fatty acids (PUFA)					Total saturated fatty acids	Total unsaturated fatty acids
		C16:0	C18:0	C20:0	C18:1n9c	C20:1	C18:2n6c	C18:3n3	C20:4n6	C20:5n3	C22:6n3		
	WR	17.77 ± 0.21^d^	1.56 ± 0.02^d^	1.93 ± 0.13^b^	40.83 ± 0.41^c^	0.64 ± 0.01^b^	18.38 ± 0.21^d^	18.83 ± 0.11^e^	0.02 ± 0.01^e^	0.02 ± 0.01^c^	0.02 ± 0.01^c^	21.26 ± 0.12^e^	78.74 ± 0.37^d^
Whole-grain (WG)	BR	15.64 ± 0.20^^e^^	3.76 ± 0.26^ab^	1.45 ± 0.12^c^	36.76 ± 0.42^e^	0.69 ± 0.03^b^	22.42 ± 0.22^c^	19.13 ± 0.12^d^	0.05 ± 0.01^e^	0.05 ± 0.02^c^	0.05 ± 0.02^c^	20.85 ± 0.33^e^	79.15 ± 0.36^c^
	ZP^1^	10.39 ± 0.15^h^	1.25 ± 0.11^e^	1.14 ± 0.12^d^	38.73 ± 0.41^d^	0.72 ± 0.01^b^	23.79 ± 0.11^a^	20.91 ± 0.21^c^	1.69 ± 0.06^c^	0.64 ± 0.11^b^	0.74 ± 0.12^b^	12.78 ± 0.25^fg^	87.22 ± 0.35^a^
	ZP^2^	10.32 ± 0.27^h^	1.28 ± 0.11^e^	1.17 ± 0.09^d^	38.41 ± 0.41^d^	0.72 ± 0.01^b^	23.38 ± 0.16^b^	21.58 ± 0.17^b^	1.73 ± 0.06^c^	0.67 ± 0.11^b^	0.74 ± 0.11^b^	12.77 ± 0.28^fg^	87.23 ± 0.26^a^
	ZP^3^	10.38 ± 0.18^h^	1.45 ± 0.13^de^	1.15 ± 0.12^d^	38.34 ± 0.41^d^	0.73 ± 0.07^b^	23.17 ± 0.11^b^	21.63 ± 0.11^b^	1.73 ± 0.03^c^	0.68 ± 0.11^b^	0.74 ± 0.11^b^	12.98 ± 0.17^fg^	87.02 ± 0.44^ab^
Milled-grain (MG)	BR	18.78 ± 0.19^c^	1.60 ± 0.03^d^	1.90 ± 0.14^b^	40.43 ± 0.64^c^	0.63 ± 0.01^b^	18.33 ± 0.11^d^	18.29 ± 0.17^f^	0.02 ± 0.01^e^	0.01 ± 0.01^c^	0.01 ± 0.01^c^	22.28 ± 0.21^d^	77.72 ± 0.29^e^
	ZP^1^	11.87± 0.11^f^	0.24 ± 0.01^f^	1.13 ± 0.21^d^	43.83 ± 0.31^a^	0.62 ± 0.01^b^	22.26 ± 0.20^c^	17.31 ± 0.13^h^	1.42 ± 0.02^d^	0.63 ± 0.12^b^	0.69 ± 0.13^b^	13.24 ± 0.22^f^	86.76 ± 0.42^b^
	ZP^2^	11.25± 0.18^g^	0.26 ± 0.02^f^	1.20 ± 0.23^d^	42.77 ± 0.22^b^	0.66 ± 0.09^b^	23.16 ± 0.14^b^	17.87 ± 0.12^g^	1.44 ± 0.08^d^	0.67 ± 0.11^b^	0.72 ± 0.23^b^	12.71 ± 0.25^g^	87.29 ± 0.22^a^
	ZP^3^	11.38± 0.12^g^	0.27 ± 0.01^f^	1.27 ± 0.13^cd^	42.61 ± 0.12^b^	0.68 ± 0.01^b^	23.16 ± 0.20^b^	17.92 ± 0.18^g^	1.51 ± 0.11^d^	0.58 ± 0.15^b^	0.62 ± 0.12^b^	12.92 ± 0.15^fg^	87.08 ± 0.22^a^^b^
Bran-powder (BP)	BR	24.04 ± 0.10^a^	3.92 ± 0.21^a^	2.28 ± 0.14^a^	32.03 ± 0.21^g^	0.94 ± 0.11^a^	13.69 ± 0.12^ef^	22.95 ± 0.21^a^	0.07 ± 0.02^e^	0.04 ± 0.02^c^	0.04 ± 0.17^c^	30.24 ± 0.19^a^	69.76 ± 0.98^h^
	ZP^1^	19.34 ± 0.21^b^	3.48 ± 0.22^c^	2.25 ± 0.11^a^	33.14 ± 0.25^f^	0.72 ± 0.13^b^	13.97 ± 0.12^e^	23.01 ± 0.22^a^	2.11 ± 0.12^b^	0.89 ± 0.06^a^	1.09 ± 0.08^a^	25.07 ± 0.38^bc^	74.93 ± 0.87^fg^
	ZP^2^	19.31 ± 0.14^b^	3.42 ± 0.24^c^	2.19 ± 0.12^a^	33.06 ± 0.32^f^	0.74 ± 0.11^b^	13.90 ± 0.19^e^	23.11 ± 0.22^a^	2.23 ± 0.10^a^^b^	0.94 ± 0.08^a^	1.10 ± 0.09^a^	24.92 ± 0.35^c^	75.08 ± 0.59^f^
	ZP^3^	19.54 ± 0.18^b^	3.62 ± 0.22^bc^	2.21 ± 0.11^a^	32.93 ± 0.24^f^	0.75 ± 0.11^b^	13.52 ± 0.18^f^	23.14 ± 0.23^a^	2.25 ± 0.12^a^	0.94 ± 0.10^a^	1.10 ± 0.11^a^	25.37 ± 0.29^b^	74.63 ± 0.87^g^

Data are represented as the mean ± standard deviation (*n* = 3). Different lowercase superscripts (a-h) in the same column indicate significant differences among samples (*P* < 0.05). The grain fractions include whole grain (WG), milled grain (MG), and bran powder (BP). The fatty acids abbreviations are as follows: palmitic acid (C16:0), stearic acid (C18:0), behenic acid (C22:0), oleic acid (C18:1n9c), eicosenoic acid (C20:1), linoleic acid (C18:2n6c), α-linolenic acid (C18:3n3), arachidonic acid (C20:4n6), eicosapentaenoic acid (C20:5n3), docosahexaenoic acid (C22:6n3). ZP^1^, ZP^2^, and ZP^3^ indicate different cultivars of wild rice (*Zizania palustris*, ZP), classified by kernel length as long (> 15 mm), medium (10–15 mm), and short (< 10 mm), respectively.

A notable finding was the substantially higher content of polyunsaturated fatty acids (PUFAs) in ZP, particularly linoleic acid (C18:2n6c) and α-linolenic acid (C18:3n3). Furthermore, a unique and potentially significant finding was the presence of detectable amounts of longer-chain PUFAs in ZP, such as arachidonic acid (C20:4n6), eicosapentaenoic acid (C20:5n3), and docosahexaenoic acid (C22:6n3). These very-long-chain PUFAs are uncommon in most cereals from the Poaceae family due to the absence of key desaturase enzymes (e.g., Δ6-desaturase) required for their biosynthesis. Their presence in ZP indicates a potentially unique fatty acid metabolism, warranting further genomic and biochemical investigation (Sayanova et al., 2011).

Milling reduced total fatty acid content but did not drastically alter the relative profile. Notably, even after milling, the milled ZP (MG-ZP) maintained a high UFA proportion (∼87%), similar to its whole grain form. As expected, the bran fraction (BP) retained the highest total fatty acid content. The absolute SFA content in ZP bran was approximately 25.12% lower than in BR bran (16.93%), which may contribute to enhanced oxidative stability of ZP during storage and processing (Fu et al., 2025). Furthermore, ZP bran also contained a high proportion of PUFAs, including the essential fatty acid α-linolenic acid (C18:3n3) and very long-chain PUFAs (e.g., C20:5n3 and C22:6n3). The levels of these specific PUFAs in ZP bran were significantly higher than those found in BR bran (Fu et al., 2025; Huang & Lai, 2016).

The results summarized in Table 4 show that, although ZP had a lower total fatty acid content compared to conventional rice, it possessed significantly higher proportions and nutritionally valuable unsaturated fatty acids (MUFAs and PUFAs) with the presence of unique long-chain fatty acids. These nutritional advantages were particularly evident in the bran fraction.

### 3.7 Total phenolic and flavonoid content

**Fig. 4:**
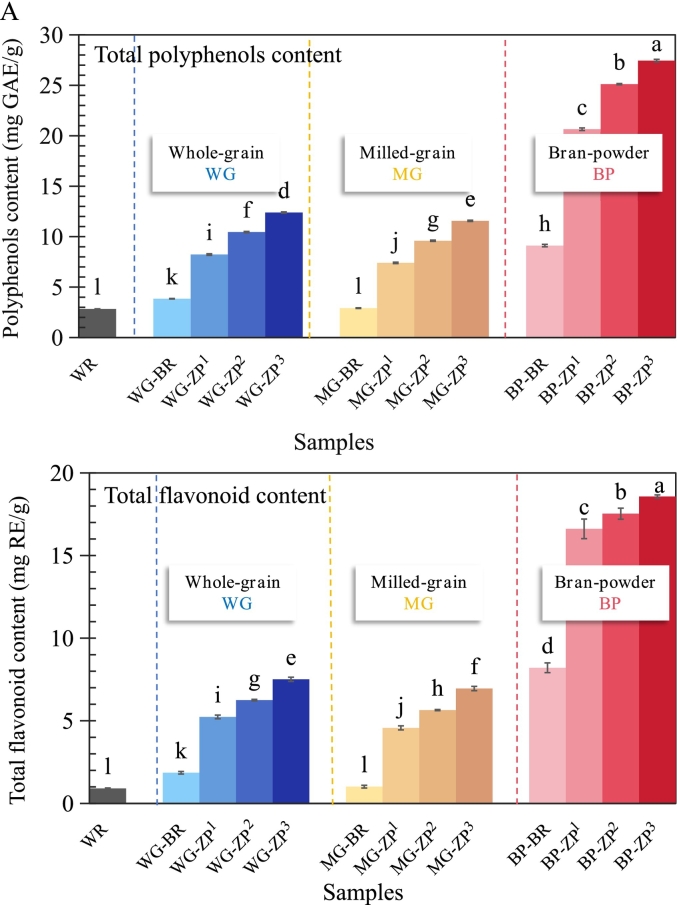

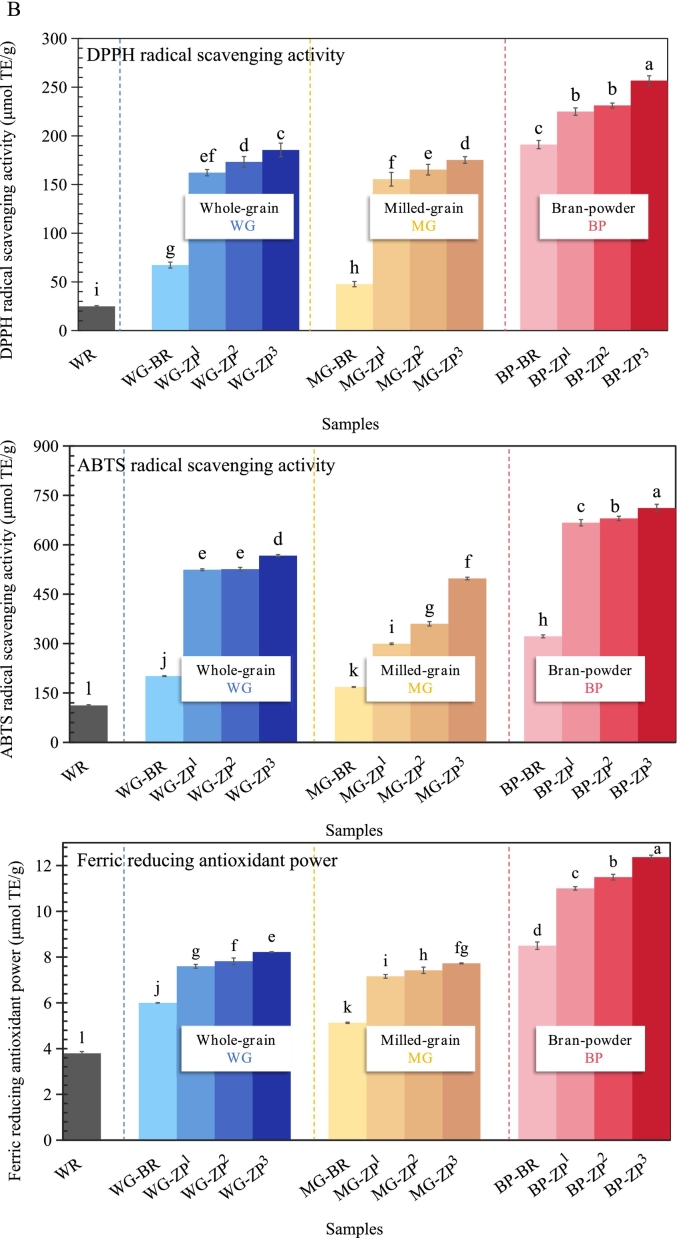
Bioactive compounds (A) and antioxidant activities (B) of whole grain (WG), milled grain (MG), and bran powder (BP) fractions from white rice (WR), brown rice (BR), and wild rice (*Zizania palustris*) cultivars (ZP^1^, ZP^2^, ZP^3^). (A) presents the total phenolic content and total flavonoid content, while panel (B) presents the antioxidant activities, including DPPH and ABTS radical scavenging capacity and ferric reducing antioxidant power (FRAP). The wild rice cultivars (ZP^1^, ZP^2^, and ZP^3^) are classified by kernel length: long (> 15 mm), medium (10–15 mm), and short (< 10 mm), respectively. (For interpretation of the references to colour in this figure legend, the reader is referred to the web version of this article.)

The total phenolic content (TPC) and total flavonoid content (TFC) varied significantly among the different rice types (WR, BR, ZP) and their fractions (WG, MG, BP), as presented in Fig. 4A. Notably, ZP exhibited significantly higher levels of TPC and TFC than the conventional rice controls, particularly in the bran fraction.

In the WG fraction, the average TPC and TFC of ZP (10.36 mg GAE/g and 6.45 mg RE/g, respectively) were approximately 2.7- to 3.7-fold higher than those of the rice controls (*P* < 0.05). These results provide definitive chemical validation for the earlier morphological observations (Sections 3.1 and 3.2), indicating that the darker and thicker bran of ZP corresponds to a higher accumulation of phenolic and flavonoid compounds (Massaretto et al., 2023). Among ZP cultivars, ZP^3^ exhibited the highest TPC and TFC, followed by ZP^2^ and ZP^1^ (*P* < 0.05). Notably, the TPC and TFC values of ZP were approximately 2–3 times higher than those reported for other pigmented whole grains, including black rice and red rice (Melini & Acquistucci, 2017). These values are also considerably higher than those recently reported for commercial wild rice (*Zizania aquatica*) by Massaretto et al. (2023), confirming the superior phenolic profile of *Zizania palustris*.

The milling process substantially reduced TPC and TFC in all samples. However, a key finding was that the milled ZP still retained higher phytochemical levels than milled BR. The calculated retention rates for TPC and TFC in milled ZP were approximately 93% and 90%, respectively, substantially higher than the 76% and 54% observed for milled BR. This notable retention of phytochemicals in milled ZP could be attributed to two factors: (i) The exceptionally high initial concentration of these compounds in the whole grain likely promotes diffusion of phenolic compounds into the aleurone layer. (ii) The unique, dense endosperm structure of ZP (Fig. 2A) may further increase retention by physically encapsulating bioactive compounds and reduce their loss during milling. This is consistent with the pronounced yellowish hue of milled ZP (Fig. 1), which contrasts sharply with milled BR. As expected, ZP bran exhibited the highest TPC and TFC levels (approximately 27.40 mg GAE/g and 18.57 mg RE/g, respectively), which were about 3.0-fold higher than those in BR bran (9.12 mg GAE/g and 8.21 mg RE/g, respectively).

Thus, ZP exhibited a nutritionally superior phenolic and flavonoid profile, which was most concentrated in the bran fraction, with ZP^3^ being the highest-yielding cultivar. These findings provide chemical validation for the morphological characteristics described in earlier sections (3.1–3.3), showing the correlation between the dark colour and substantial thickness of ZP bran and its high content of bioactive compounds.

### 3.8 DPPH, ABTS, and FRAP antioxidant activities

The DPPH radical scavenging activity, ABTS radical scavenging activity, and ferric reducing antioxidant power (FRAP) of WR, BR, and ZP are shown in Fig. 4B. Consistent with the phytochemical results, ZP showed a markedly higher antioxidant capacity in all three assays (DPPH, ABTS, FRAP) compared to conventional rice.

In the WG fraction, WR exhibited the lowest antioxidant activities, with values of 24.90 (DPPH), 112.12 (ABTS), and 3.79 μmol TE/g (FRAP), while the corresponding values for BR were 67.30, 201.19, and 6.00 μmol TE/g, respectively. In contrast, the ZP cultivars showed significantly higher antioxidant activities. The antioxidant activities of ZP were 2.1- to 7.0-fold higher than those of WR, and 1.3- to 2.7-fold higher than BR. This magnitude of increase is consistent with findings from Yang et al. (2025), who reported similarly enhanced antioxidant capacity in wild rice populations from Canada. The magnitude of increase in antioxidant activity (up to 7-fold) exceeded the increase in TPC/TFC (2.7–3.7 fold), suggesting a synergistic contribution from non-phenolic antioxidants. These were associated with their high contents of total phenolics and flavonoids (Section 3.7), the unique darker bran pigmentation (Sections 3.1 and 3.2), and high levels of mineral content (Section 3.5). This integrated profile in ZP contributes to its enhanced antioxidant capacity. The high contents of phenolic and flavonoid compounds function as direct free radical scavengers, which can be supported by the deep colour of bran. Furthermore, the high levels of minerals such as manganese, copper, and zinc in ZP also serve as essential cofactors for endogenous antioxidant enzymes, thereby reinforcing its overall antioxidant activity (Marques, 2024; Wei et al., 2024).

Milling led to a reduction in antioxidant activity, but the degree of loss was significantly lower in ZP than in BR. The DPPH, ABTS, and FRAP values for milled BR decreased by 14.5–29.1% compared to its whole grain form, whereas the corresponding decreases for milled ZP were only 5.5–12.2%. For example, the DPPH activity of milled ZP (155.43–175.23 μmol TE/g) was approximately 3.5-fold higher than that of milled BR (47.70 μmol TE/g). As previously noted, this resilience to processing is directly linked to the higher retention of phenolic compounds (Section 3.7) and is consistent with the proposed mechanisms of deeper phytochemical distribution and physical encapsulation within the ZP kernel. Among all fractions, ZP bran exhibited the highest antioxidant activity. The ABTS, DPPH, and FRAP values for ZP bran were 1.5-, 2.1-, and 2.6-fold higher, respectively, than those of BR bran (*P* < 0.05). This indicates that the intense pigmentation of ZP bran is a direct indicator of its potent antioxidant reservoir.

Overall, ZP cultivars exhibited significantly higher antioxidant capacity compared to conventional rice varieties, with the bran fraction showing the highest activity, and the antioxidant activity was notably well-retained after milling. Among ZP cultivars, ZP^3^ exhibited the highest antioxidant activity, likely attributable to its genotype-dependent biosynthesis of antioxidant compounds (e.g., phenolic and flavonoid) and its darker pericarp phenotype, which is associated with higher accumulation of these bioactive substances. The superior antioxidant capacity of ZP is a multifactorial trait arising from: (i) high concentrations of primary antioxidant phenolics and flavonoids; (ii) a supporting matrix of antioxidant mineral cofactors; and (iii) a unique kernel morphology that enhances the retention of these protective compounds during processing. This integrated antioxidant profile is most concentrated in the bran but remains robust in the milled grain, highlighting the functional value of ZP across different product forms.

## 4 Conclusions

This study comprehensively compared the nutritional composition and functional properties of wild rice (*Zizania palustris*, ZP) and conventional rice varieties in whole grain, milled grain, and bran fractions. Wild rice contained significantly higher levels of protein, dietary fiber, essential minerals, phenolic and flavonoid compounds, along with a more balanced amino acid profile (close to FAO/WHO recommendations) and higher antioxidant capacity. A notable finding was the presence of long-chain PUFAs in ZP, which are uncommon in most cereal grains. Although milling reduced nutrient content in all samples, the milled ZP grain retained substantially higher antioxidant activity compared to BR, and the bran fraction consistently showed the highest concentration of nutrients and bioactivity. The combination of lower starch content with higher dietary fiber and protein content supports the potential of ZP as a suitable ingredient for staple foods development. From an industrial perspective, the bran fraction serves as a concentrated source of natural antioxidants and functional components for dietary supplements or nutritional fortification of cereal-based products (e.g., blended flours, snack bars). The whole grain offers a balanced nutrient profile suitable for health-conscious consumers, while the milled fraction may serve as a milder base for conventional rice products. Future research should focus on optimizing processing techniques to preserve nutritional integrity, enhance the bioavailability of bioactive compounds, and improve consumer acceptance to facilitate broader application.

## CRediT authorship contribution statement

**Xin-Yu Li:** Writing – original draft, Methodology, Investigation. **Yi-Tong Zhang:** Methodology, Investigation. **Yu-Jie Wang:** Methodology, Investigation. **Sheng-Yi Wang:** Methodology, Investigation. **Yong-Li Wang:** Methodology, Investigation. **Ya-Li Liu:** Methodology, Investigation. **Chu-Yun Wu:** Methodology, Investigation. **Jian-Ya Qian:** Supervision, Formal analysis. **Chen Zhang:** Writing – review & editing, Writing – original draft, Funding acquisition, Formal analysis, Conceptualization.

## Declaration of competing interest

The authors declare that they have no known competing financial interests or personal relationships that could have appeared to influence the work reported in this paper.

## Data Availability

Data will be made available on request.
