# Supplementary material for: Exploring the functional food potential of wild rice (Zizania palustris): A comparative analysis of morphological, nutritional, bioactive compounds, and antioxidant activity in whole, milled, and bran fractions
Source: Food Chem X. 2026 May 8;36:103961. doi: 10.1016/j.fochx.2026.103961 (PMC13195364; doi:10.1016/j.fochx.2026.103961)
Supplement: Supplementary file 1 — Supplementary material [file mmc1.docx]

**Supplementary Material Table S1.** Designation and nomenclature of experimental samples

| Experimental design | Sample type | Nomenclature |
| --- | --- | --- |
|  | White rice | WR |
| Whole-grain (WG) | Brown rice | WG-BR |
|  | Wild rice (*Zizania palustris* No.1, No.2, No.3 classified by kernel length) | WG-ZP^1^, WG-ZP^2^, WG-ZP^3^ |
| Milled-grain (MG) | Brown rice | MG-BR |
|  | Wild rice (*Zizania palustris* No.1, No.2, No.3 classified by kernel length) | MG-ZP^1^, MG-ZP^2^, MG-ZP^3^ |
| Bran-powder (BP) | Brown rice | BP-BR |
|  | Wild rice *(Zizania palustris* No.1, No.2, No.3 classified by kernel length) | BP-ZP^1^, BP-ZP^2^, BP-ZP^3^ |
